# 3-Carboxy­methyl-1,3-benzimidazolium-1-acetate monohydrate

**DOI:** 10.1107/S1600536809027391

**Published:** 2009-07-25

**Authors:** Jinling Miao, Yong Nie, Hongwei Chen, Jingtao Li, Daqi Wang

**Affiliations:** a School of Chemistry and Chemical Engineering, University of Jinan, Jinan 250022, People’s Republic of China; b College of Chemistry and Chemical Engineering, Liaocheng University, Liaocheng 252059, People’s Republic of China.

## Abstract

The title compound, C_11_H_10_N_2_O_4_·H_2_O, has a zwitterionic structure, in which the benzimidazole ring system is planar, with a maximum deviation of 0.007 (3) Å. The carbox­yl/carboxyl­ate groups adopt a *trans* configuration. In the crystal structure, inter­molecular O—H⋯O hydrogen bonds involving the hydr­oxy/oxide O atoms link the mol­ecules into a one-dimensional chain. These chains are further linked by O—H⋯O hydrogen bonds involving the water mol­ecules into a two-dimensional network. π–π contacts between the benzimidazole rings [centroid–centroid distance = 3.5716 (4) Å] lead to the formation of a three-dimensional supra­molecular structure.

## Related literature

For a related structure, see: Chen & Huang (2006 ▶).
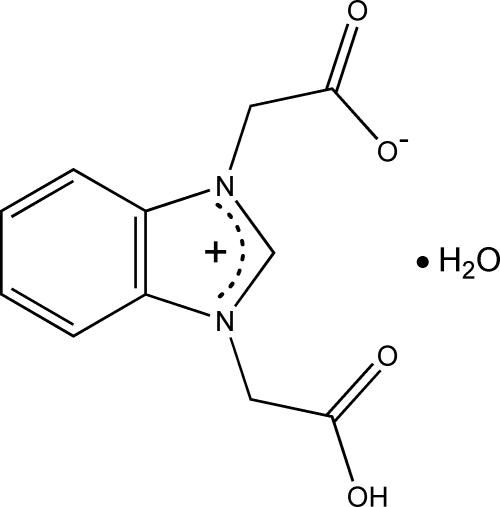


## Experimental

### 

#### Crystal data

C_11_H_10_N_2_O_4_·H_2_O
*M* *_r_* = 252.23
Monoclinic, 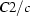

*a* = 16.0731 (15) Å
*b* = 8.1619 (11) Å
*c* = 18.8678 (17) Å
β = 113.3680 (10)°
*V* = 2272.2 (4) Å^3^
*Z* = 8
Mo *K*α radiation
μ = 0.12 mm^−1^
*T* = 298 (2) K
0.50 × 0.40 × 0.20 mm

#### Data collection

Bruker SMART 1000 CCD area-detector diffractometer
Absorption correction: multi-scan (*SADABS*; Bruker, 2001 ▶) *T*_min_ = 0.943, *T*_max_ = 0.977
5875 measured reflections
2213 independent reflections
1495 reflections with *I* > 2σ(*I*)
*R*_int_ = 0.048

#### Refinement

*R*[*F* ^2^ > 2σ(*F* ^2^)] = 0.041
*wR*(*F* ^2^) = 0.120
*S* = 1.00
2213 reflections
173 parameters
1 restraint
H atoms treated by a mixture of independent and constrained refinement
Δρ_max_ = 0.25 e Å^−3^
Δρ_min_ = −0.18 e Å^−3^

### 

Data collection: *SMART* (Bruker, 2001 ▶); cell refinement: *SAINT* (Bruker, 2001 ▶); data reduction: *SAINT*; program(s) used to solve structure: *SHELXS97* (Sheldrick, 2008 ▶); program(s) used to refine structure: *SHELXL97* (Sheldrick, 2008 ▶); molecular graphics: *SHELXTL* (Sheldrick, 2008 ▶); software used to prepare material for publication: *SHELXTL*.

## Supplementary Material

Crystal structure: contains datablocks I, global. DOI: 10.1107/S1600536809027391/hk2729sup1.cif

Structure factors: contains datablocks I. DOI: 10.1107/S1600536809027391/hk2729Isup2.hkl

Additional supplementary materials:  crystallographic information; 3D view; checkCIF report

## Figures and Tables

**Table 1:** Hydrogen-bond geometry (Å, °)

*D*—H⋯*A*	*D*—H	H⋯*A*	*D*⋯*A*	*D*—H⋯*A*
O3—H3⋯O2^i^	0.925 (17)	1.592 (18)	2.487 (2)	162 (2)
O5—H5⋯O1	0.88 (3)	1.95 (3)	2.825 (2)	172 (3)
O6—H6⋯O1	0.84 (3)	2.11 (3)	2.943 (2)	173 (3)

Symmetry code: (i) 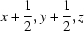.

**Fig. 1.**
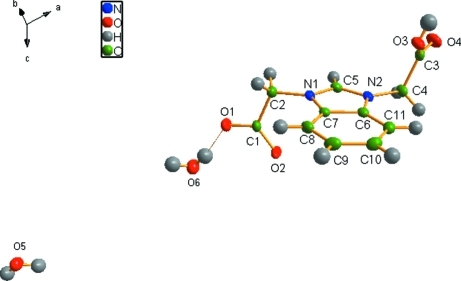
The molecular structure of the title molecule, with the atom-numbering scheme. Displacement ellipsoids are drawn at the 25% probability level.

**Fig. 2.**
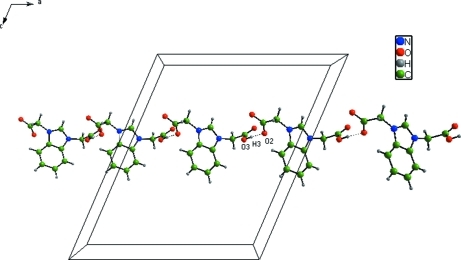
The one-dimensional chain constructed by hydrogen bondings.

**Fig. 3.**
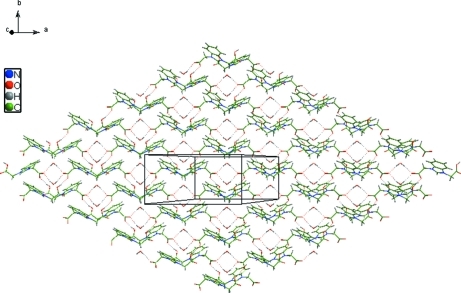
The two-dimensional network viewed along [001] direction.

**Fig. 4.**
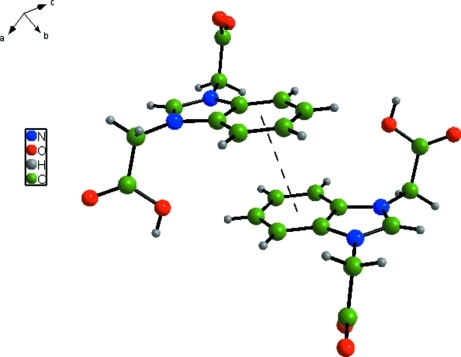
The π-π stacking between the benzene rings.

**Fig. 5.**
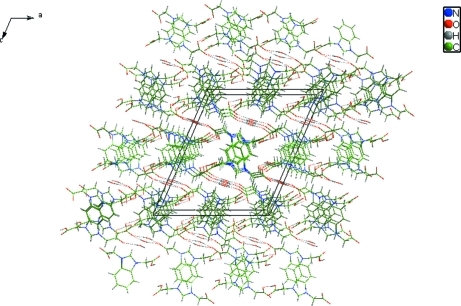
The three-dimensional network viewed along the b axis.

## supplementary crystallographic information

### Comment

Benzimidazole carboxylic acids have received much attention because of their application in the design of therapeutic agents and in construction of supramolecular metal complexes. Previously, the synthesis and crystal structure of 1-(carboxymethyl)-1,3-benzimidazol-3-ium-3-acetate, (II), have been described (Chen & Huang, 2006). We report herein the crystal struture of the title compound, (I).

In the molecule of the title compound (Fig. 1), the benzimidazole ring system is planar with a maximum deviation of 0.007 (3) Å for atom N1, and the two carboxyl groups adopt a *trans* configuration with respect to the benzimidazole ring plane. The C—N bonds on the imidazolium rings are found to be within 1.323 (2)–1.391 (2) Å, which are between the C—N single and C=N double bonds, suggesting charge delocalization on the imidazolium rings. The torsion angles of C5—N1—C2—C1 [95.5 (2)°] and C6—N2—C4—C3 [-89.5 (2)°] are much smaller than the corresponding values in (II). The lattice water molecules have site symmetries 2.

In the crystal structure, intermolecular O-H···O hydrogen bonds involving the hydroxy O atoms (Table 1) link the molecules into a one-dimensional chain (Fig. 2), in which they are further linked by O-H···O hydrogen bonds of lattice water molecules (Table 1) into a two-dimensional network (Fig. 3). The π···π contacts between the benzene rings of the benzimidazole groups (Fig. 4), Cg2···Cg2^i^ [symmetry code: (i) -x, 2 - y, -z, where Cg2 is centroid of the ring (C6-C11)] may further stabilize the structure, with centroid-centroid distance of 3.5716 (4) Å and lead to the formation of a three-dimensional supramolecular structure (Fig. 5).

### Experimental

For the preparation of the title compound, benzimidazole (0.714 g,6 mmol) was added to an aqueous solution (35 ml) of iodoacetic acid (1.859 g, 10 mmol) and NaOH (0.405 g, 10 mmol). The resulting mixture was heated at reflux during which benzimidazole was gradually dissolved and the colorless solution changed to yellow. The pH was adjusted using saturated NaOH solution at 20 min intervals, keeping in the range of 8–9. When no pH change was detected, the solution was further refluxed for 30 min, cooled, acidified with hydrochloric acid until pH = 2–3. The brown precipitate formed was filtered and recrystallized using water during which the deep yellow solution changed to colorless. The colorless plate crystals were formed after 5 d.

### Refinement

Atoms H3, H5 and H6 were located in a difference Fourier map and refined isotropically. The remaining H atoms were positioned geometrically with C-H = 0.93 and 0.97 Å for aromatic and methylene H atoms, respectively, and constrained to ride on their parent atoms, with U_iso_(H) = 1.2U_eq_(C).

### Crystal data

**Table:**

C_11_H_10_N_2_O_4_·H_2_O	*F*(000) = 1056
*M**_r_* = 252.23	*D*_x_ = 1.475 Mg m^−^^3^
Monoclinic, *C*2/*c*	Mo *K*α radiation, λ = 0.71073 Å
Hall symbol: -C 2yc	Cell parameters from 1932 reflections
*a* = 16.0731 (15) Å	θ = 2.4–26.4°
*b* = 8.1619 (11) Å	µ = 0.12 mm^−^^1^
*c* = 18.8678 (17) Å	*T* = 298 K
β = 113.368 (1)°	Plate, colorless
*V* = 2272.2 (4) Å^3^	0.50 × 0.40 × 0.20 mm
*Z* = 8	

### Data collection

**Table:**

Bruker SMART 1000 CCD area-detector diffractometer	2213 independent reflections
Radiation source: fine-focus sealed tube	1495 reflections with *I* > 2σ(*I*)
graphite	*R*_int_ = 0.048
φ and ω scans	θ_max_ = 26.0°, θ_min_ = 2.4°
Absorption correction: multi-scan (*SADABS*; Bruker, 2001)	*h* = −14→19
*T*_min_ = 0.943, *T*_max_ = 0.977	*k* = −10→10
5875 measured reflections	*l* = −23→23

### Refinement

**Table:**

Refinement on *F*^2^	Primary atom site location: structure-invariant direct methods
Least-squares matrix: full	Secondary atom site location: difference Fourier map
*R*[*F*^2^ > 2σ(*F*^2^)] = 0.041	Hydrogen site location: inferred from neighbouring sites
*wR*(*F*^2^) = 0.120	H atoms treated by a mixture of independent and constrained refinement
*S* = 1.00	*w* = 1/[σ^2^(*F*_o_^2^) + (0.0647*P*)^2^ + 0.9016*P*] where *P* = (*F*_o_^2^ + 2*F*_c_^2^)/3
2213 reflections	(Δ/σ)_max_ = 0.001
173 parameters	Δρ_max_ = 0.25 e Å^−^^3^
1 restraint	Δρ_min_ = −0.18 e Å^−^^3^

### Special details

**Table:**

Refinement. Refinement of F^2^ against ALL reflections. The weighted *R*-factor *wR* and goodness of fit S are based on F^2^, conventional *R*-factors *R* are based on F, with F set to zero for negative F^2^. The threshold expression of F^2^ > 2sigma(F^2^) is used only for calculating *R*-factors(gt) *etc*. and is not relevant to the choice of reflections for refinement. *R*-factors based on F^2^ are statistically about twice as large as those based on F, and R– factors based on ALL data will be even larger.

### Fractional atomic coordinates and isotropic or equivalent isotropic displacement parameters (Å^2^)

**Table:**

	*x*	*y*	*z*	*U*_iso_*/*U*_eq_	
O1	0.64155 (9)	0.6138 (2)	0.28621 (9)	0.0608 (5)	
O2	0.75932 (9)	0.5077 (2)	0.38158 (10)	0.0658 (6)	
O3	1.16479 (10)	0.80460 (19)	0.41440 (9)	0.0501 (4)	
H3	1.1979 (16)	0.867 (3)	0.3937 (14)	0.075*	
O4	1.20178 (11)	0.6063 (2)	0.35149 (10)	0.0646 (5)	
O5	0.5000	0.8452 (3)	0.2500	0.0582 (6)	
H5	0.5476 (18)	0.782 (4)	0.2614 (17)	0.087*	
O6	0.5000	0.3611 (3)	0.2500	0.0688 (8)	
H6	0.544 (2)	0.427 (4)	0.2634 (19)	0.103*	
N1	0.87906 (10)	0.7126 (2)	0.36403 (9)	0.0349 (4)	
N2	1.01818 (10)	0.62393 (18)	0.40836 (9)	0.0337 (4)	
C1	0.72276 (12)	0.6073 (3)	0.32812 (11)	0.0377 (5)	
C2	0.78365 (12)	0.7326 (3)	0.31305 (11)	0.0416 (5)	
H2A	0.7772	0.7230	0.2599	0.050*	
H2B	0.7641	0.8417	0.3199	0.050*	
C3	1.16099 (12)	0.6581 (3)	0.38815 (11)	0.0372 (5)	
C4	1.10076 (13)	0.5451 (3)	0.41022 (13)	0.0411 (5)	
H4A	1.0840	0.4524	0.3752	0.049*	
H4B	1.1348	0.5034	0.4618	0.049*	
C5	0.94014 (13)	0.6308 (2)	0.34744 (11)	0.0376 (5)	
H5A	0.9297	0.5845	0.2996	0.045*	
C6	1.00799 (12)	0.7058 (2)	0.46895 (11)	0.0322 (4)	
C7	0.91917 (12)	0.7614 (2)	0.44087 (10)	0.0320 (4)	
C8	0.88646 (14)	0.8494 (3)	0.48694 (12)	0.0429 (5)	
H8	0.8271	0.8877	0.4683	0.052*	
C9	0.94620 (17)	0.8773 (3)	0.56172 (13)	0.0494 (6)	
H9	0.9265	0.9350	0.5947	0.059*	
C10	1.03527 (16)	0.8218 (3)	0.58943 (12)	0.0480 (6)	
H10	1.0736	0.8443	0.6403	0.058*	
C11	1.06811 (14)	0.7353 (3)	0.54415 (11)	0.0409 (5)	
H11	1.1277	0.6981	0.5628	0.049*	

### Atomic displacement parameters (Å^2^)

**Table:**

	*U*^11^	*U*^22^	*U*^33^	*U*^12^	*U*^13^	*U*^23^
O1	0.0289 (8)	0.0773 (13)	0.0644 (10)	−0.0032 (8)	0.0059 (7)	0.0195 (9)
O2	0.0296 (8)	0.0579 (11)	0.0966 (13)	−0.0030 (7)	0.0107 (8)	0.0402 (10)
O3	0.0451 (9)	0.0378 (10)	0.0757 (11)	−0.0041 (7)	0.0328 (8)	0.0023 (8)
O4	0.0649 (11)	0.0641 (12)	0.0884 (12)	0.0002 (9)	0.0552 (10)	−0.0062 (9)
O5	0.0550 (15)	0.0519 (16)	0.0612 (14)	0.000	0.0161 (13)	0.000
O6	0.0574 (16)	0.0520 (17)	0.0872 (19)	0.000	0.0181 (15)	0.000
N1	0.0279 (8)	0.0388 (10)	0.0393 (9)	−0.0036 (7)	0.0147 (7)	0.0031 (7)
N2	0.0306 (9)	0.0294 (9)	0.0451 (9)	−0.0026 (7)	0.0193 (8)	−0.0003 (7)
C1	0.0250 (10)	0.0426 (13)	0.0433 (11)	0.0014 (9)	0.0111 (9)	0.0025 (10)
C2	0.0323 (11)	0.0449 (13)	0.0440 (11)	0.0004 (10)	0.0115 (9)	0.0092 (10)
C3	0.0276 (10)	0.0401 (13)	0.0428 (11)	0.0054 (9)	0.0129 (9)	0.0027 (10)
C4	0.0383 (11)	0.0327 (12)	0.0576 (12)	0.0032 (9)	0.0246 (10)	0.0002 (10)
C5	0.0378 (11)	0.0383 (12)	0.0410 (11)	−0.0087 (9)	0.0203 (9)	−0.0027 (9)
C6	0.0343 (10)	0.0242 (10)	0.0419 (11)	−0.0019 (8)	0.0191 (9)	0.0028 (9)
C7	0.0318 (10)	0.0281 (11)	0.0391 (10)	−0.0016 (8)	0.0172 (8)	0.0060 (8)
C8	0.0441 (12)	0.0381 (13)	0.0551 (13)	0.0084 (10)	0.0287 (11)	0.0078 (10)
C9	0.0702 (16)	0.0377 (13)	0.0503 (13)	0.0032 (11)	0.0345 (12)	−0.0009 (10)
C10	0.0603 (15)	0.0413 (13)	0.0389 (11)	−0.0061 (11)	0.0158 (11)	−0.0014 (10)
C11	0.0379 (11)	0.0351 (12)	0.0457 (12)	−0.0012 (9)	0.0124 (10)	0.0051 (10)

### Geometric parameters (Å, °)

**Table:**

O1—C1	1.230 (2)	C2—H2B	0.9700
O2—C1	1.246 (2)	C3—C4	1.511 (3)
O3—C3	1.286 (3)	C4—H4A	0.9700
O3—H3	0.925 (17)	C4—H4B	0.9700
O4—C3	1.203 (2)	C5—H5A	0.9300
O5—H5	0.88 (3)	C6—C11	1.385 (3)
O6—H6	0.84 (3)	C6—C7	1.387 (3)
N1—C5	1.323 (2)	C7—C8	1.382 (3)
N1—C7	1.391 (2)	C8—C9	1.375 (3)
N1—C2	1.461 (2)	C8—H8	0.9300
N2—C5	1.324 (2)	C9—C10	1.391 (3)
N2—C6	1.389 (2)	C9—H9	0.9300
N2—C4	1.463 (2)	C10—C11	1.366 (3)
C1—C2	1.519 (3)	C10—H10	0.9300
C2—H2A	0.9700	C11—H11	0.9300
			
C3—O3—H3	107.1 (16)	C3—C4—H4B	108.9
C5—N1—C7	108.08 (16)	H4A—C4—H4B	107.7
C5—N1—C2	125.76 (17)	N1—C5—N2	110.62 (17)
C5—N2—C6	108.28 (15)	N1—C5—H5A	124.7
C5—N2—C4	125.26 (16)	N2—C5—H5A	124.7
C6—N2—C4	126.45 (16)	C11—C6—C7	121.93 (18)
C7—N1—C2	125.85 (16)	C11—C6—N2	131.65 (18)
O1—C1—O2	126.00 (19)	C7—C6—N2	106.41 (16)
O1—C1—C2	116.68 (18)	C8—C7—C6	121.35 (18)
O2—C1—C2	117.31 (16)	C8—C7—N1	132.04 (18)
N1—C2—C1	112.79 (16)	C6—C7—N1	106.60 (16)
N1—C2—H2A	109.0	C9—C8—C7	116.5 (2)
C1—C2—H2A	109.0	C9—C8—H8	121.7
N1—C2—H2B	109.0	C7—C8—H8	121.7
C1—C2—H2B	109.0	C8—C9—C10	121.8 (2)
H2A—C2—H2B	107.8	C8—C9—H9	119.1
O4—C3—O3	126.48 (19)	C10—C9—H9	119.1
O4—C3—C4	119.9 (2)	C11—C10—C9	121.9 (2)
O3—C3—C4	113.53 (17)	C11—C10—H10	119.0
N2—C4—C3	113.53 (16)	C9—C10—H10	119.0
N2—C4—H4A	108.9	C10—C11—C6	116.4 (2)
C3—C4—H4A	108.9	C10—C11—H11	121.8
N2—C4—H4B	108.9	C6—C11—H11	121.8
			
C5—N1—C2—C1	95.5 (2)	C11—C6—C7—C8	0.1 (3)
C7—N1—C2—C1	−77.4 (2)	N2—C6—C7—C8	−179.74 (17)
O1—C1—C2—N1	−178.35 (18)	C11—C6—C7—N1	179.22 (17)
O2—C1—C2—N1	1.7 (3)	N2—C6—C7—N1	−0.61 (19)
C5—N2—C4—C3	89.6 (2)	C5—N1—C7—C8	179.7 (2)
C6—N2—C4—C3	−89.5 (2)	C2—N1—C7—C8	−6.3 (3)
O4—C3—C4—N2	−143.40 (19)	C5—N1—C7—C6	0.7 (2)
O3—C3—C4—N2	38.8 (2)	C2—N1—C7—C6	174.70 (16)
C7—N1—C5—N2	−0.5 (2)	C6—C7—C8—C9	−0.5 (3)
C2—N1—C5—N2	−174.54 (16)	N1—C7—C8—C9	−179.39 (19)
C6—N2—C5—N1	0.2 (2)	C7—C8—C9—C10	0.7 (3)
C4—N2—C5—N1	−179.06 (16)	C8—C9—C10—C11	−0.6 (3)
C5—N2—C6—C11	−179.5 (2)	C9—C10—C11—C6	0.1 (3)
C4—N2—C6—C11	−0.3 (3)	C7—C6—C11—C10	0.1 (3)
C5—N2—C6—C7	0.30 (19)	N2—C6—C11—C10	179.90 (19)
C4—N2—C6—C7	179.50 (16)		

### Hydrogen-bond geometry (Å, °)

**Table:**

*D*—H···*A*	*D*—H	H···*A*	*D*···*A*	*D*—H···*A*
O3—H3···O2^i^	0.93 (2)	1.59 (2)	2.487 (2)	162 (2)
O5—H5···O1	0.88 (3)	1.95 (3)	2.825 (2)	172 (3)
O6—H6···O1	0.84 (3)	2.11 (3)	2.943 (2)	173 (3)

Symmetry codes: (i) *x*+1/2, *y*+1/2, *z*.
